# Essential Roles for Mannose-Binding Lectin-Associated Serine Protease-1/3 in the Development of Lupus-Like Glomerulonephritis in MRL/*lpr* Mice

**DOI:** 10.3389/fimmu.2018.01191

**Published:** 2018-05-28

**Authors:** Takeshi Machida, Natsumi Sakamoto, Yumi Ishida, Minoru Takahashi, Teizo Fujita, Hideharu Sekine

**Affiliations:** ^1^Department of Immunology, Fukushima Medical University, Fukushima, Japan; ^2^Fukushima Prefectural General Hygiene Institute, Fukushima, Japan

**Keywords:** systemic lupus erythematosus, lupus nephritis, complement, MASP-1/3, lectin pathway, alternative pathway, murine models

## Abstract

The complement system, composed of the three activation pathways, has both protective and pathogenic roles in the development of systemic lupus erythematosus (or lupus), a prototypic autoimmune disease. The classical pathway contributes to the clearance of immune complexes (ICs) and apoptotic cells, whereas the alternative pathway (AP) exacerbates renal inflammation. The role of the lectin pathway (LP) in lupus has remained largely unknown. Mannose-binding lectin (MBL)-associated serine proteases (MASPs), which are associated with humoral pattern recognition molecules (MBL or ficolins), are the enzymatic constituents of the LP and AP. MASP-1 encoded by the *Masp1* gene significantly contributes to the activation of the LP. After the binding of MBL/ficolins to pathogens or self-altered cells, MASP-1 autoactivates first, then activates MASP-2, and both participate in the formation of the LP C3 convertase C4b2a, whereas, MASP-3, the splice variant of the *Masp1* gene, is required for the activation of the zymogen of factor D (FD), and finally participates in the formation of the AP C3 convertase C3bBb. To investigate the roles of MASP-1 and MASP-3 in lupus, we generated *Masp1* gene knockout lupus-prone MRL/*lpr* mice (*Masp1/3^−/−^* MRL/*lpr* mice), lacking both MASP-1 and MASP-3, and analyzed their renal disease. As expected, sera from *Masp1/3^−/−^* MRL/*lpr* mice had no or markedly reduced activation of the LP and AP with zymogen forms of complement FD. Compared to their wild-type littermates, the *Masp1/3^−/−^* MRL/*lpr* mice had maintained serum C3 levels, little-to-no albuminuria, as well as significantly reduced glomerular C3 deposition levels and glomerular pathological score. On the other hand, there were no significant differences in the levels of serum anti-dsDNA antibody, circulating ICs, glomerular IgG and MBL/ficolins deposition, renal interstitial pathological score, urea nitrogen, and mortality between the wild-type and *Masp1/3^−/−^* MRL/*lpr* mice. Our data indicate that MASP-1/3 plays essential roles in the development of lupus-like glomerulonephritis in MRL/*lpr* mice, most likely *via* activation of the LP and/or AP.

## Introduction

The complement system, which consists of over 30 soluble and membrane-bound proteins, plays protective roles in host defense and a role in some immune regulatory functions *via* activation of the three different initial complement pathways: the classical pathway (CP), lectin pathway (LP), and alternative pathway (AP) (1). Each pathway follows a sequence of reactions to generate a C3 convertase (C4b2a in the CP and LP or C3bBb in the AP), and subsequently a C5 convertase (C4b2a3b or C3bBb3b). The terminal sequence of complement activation involves C5b, C6, C7, C8, and C9, which interact sequentially to form the membrane attack complex.

Activation of the CP is initiated by the binding of a C1 complex (C1q, C1r, and C1s), in which C1q recognizes IgM or IgG of antigen (Ag)–antibody complexes, followed by the activation of C1r and C1s, subsequently C4 and C2, resulting in the creation of C3 convertase C4b2a (2). However, activation of the LP is initiated by the binding of the LP pattern recognition molecules (PRMs), such as mannose-binding lectin (MBL), ficolins (-1, -2, -3 or M-, L-, H-, respectively), collectin (CL)-10, and CL-11 (3). The function of MBL or ficolins in opsonophagocytosis and in the complement pathway is similar to that of C1q. MBL-associated serine proteases-1 and -2 (MASP-1 and MASP-2), the enzymatic constituents of the LP, form a complex with the LP PRMs. After binding of the LP PRMs to carbohydrates typically found on the surface of microorganisms, MASP-1 autoactivates first, and subsequently activates MASP-2 (4). Activated MASP-2 cleaves both C4 and C2, resulting in the creation of C3 convertase C4b2a (5). However, activated MASP-1 cleaves MASP-2 and C2 but not C4 (6). Unlike the CP and LP, initiation of the AP does not require recognition molecules, and is thought to occur by a process termed “tickover,” the spontaneous thioester hydrolysis of C3 (7). The product, C3(H_2_O), interacts with factor B (FB), and the subsequent cleavage of FB by the serine protease factor D (FD). This results in the creation of C3 convertase C3(H_2_O)Bb, which cleaves C3 generating metastable C3b. The thioester bond in metastable C3b mediates covalent attachment of C3b to the surface of self (i.e., host) or non-self (i.e., microorganisms) cell membrane. C3b bound to the host cells is subject to process inactivation by multiple complement-regulatory proteins, present in plasma and on host cell membranes. By contrast, C3b bound to microorganisms is subject to process a chain reaction-like amplification loop that can bind large numbers of C3b molecules on the cell surface after the initial C3b binding. Notably, uncontrolled activation of the AP is associated with multiple inflammatory diseases, such as systemic lupus erythematosus (SLE or lupus).

Our group previously provided a fundamental link between the LP and AP. To investigate the role for MASP-1 for complement activation, we generated C57BL/6 mice deficient for MASP-1 by targeting of the *Masp1* gene that transcribes two serine proteases, MASP-1 and MASP-3, and MAp44, which lacks a serine protease domain. In addition, MAp44 has been suggested to act as a competitive inhibitor of LP activation. Unexpectedly, our previous studies reported that *Masp1/3^−/−^* mice had little-to-no activation of both the LP and AP with an inactive form of FD (pro-FD) in their sera, indicating that MASP-1 and/or MASP-3 play essential roles in LP and AP activation (4, 8). We also reported that recombinant MASP-3 cleaved pro-FD in mouse serum (9), suggesting a role for MASP-3 in AP activation. Furthermore, a recent *in vitro* study using selective inhibitors against MASPs demonstrated that the monospecific MASP-1 inhibitor, SGMI-1, completely inhibited LP activation in human resting blood, and it was more potent than the monospecific MASP-2 inhibitor SGMI-2 (10). Another recent publication using SGMI-1 showed that MASP-1 is essential for LPS-induced but not for zymosan-induced AP activation under a Ca^2+^-chelating condition, suggesting the roles for MASP-1 in AP activation in addition to LP activation (11). On the other hand, the monospecific MASP-3 inhibitor TFMI-3 did not inhibit any of the three complement pathways in normal human serum, but completely blocked pro-FD activation in normal human plasma (12).

Systemic lupus erythematosus is a prototypic human systemic autoimmune disease involving aberrant complement activation that is initiated by immune complexes (ICs) formed by autoantibodies (auto-Abs) directed against a broad range of self Ags, including dsDNA and nuclear proteins. The kidney is a major site of IC formation and/or deposition, and lupus nephritis is a major cause of mortality in both human SLE and murine models of lupus. The roles of each complement pathway in the development of lupus nephritis have been investigated using serum and biopsy samples from lupus patients or murine models of lupus. CP activation is assumed to play a role in initial pathogenic complement activation in lupus nephritis since both lupus patients and lupus-prone mice exhibit significant glomerular IC deposition consisting of auto-Ag–auto-Ab complexes (e.g., dsDNA-anti-dsDNA Ab complexes) and C1q, a recognition molecule for the CP. Paradoxically, patients with homozygous deficiency of the CP components, including C1 (C1q, C1r, or C1s), C4, or C2, have a high risk of lupus or lupus-like disease (13, 14). Previous studies on murine models of lupus showed that mice deficient for C1q or C4 exhibited high titers of serum antinuclear Ab, thus increasing the incidence and prevalence of glomerular disease associated with multiple apoptotic bodies or lupus-like glomerulonephritis, as well as increased mortality (15–17). These results clearly indicate that the CP plays both pathogenic and protective roles against the development of lupus, including IC-mediated glomerulonephritis.

On the other hand, there is strong evidence that AP activation plays an exacerbating role in the development of lupus glomerulonephritis, either through direct initiation of the pathway or the magnifying effects of the amplification loop. Deficiency of the AP components in lupus-prone MRL/*lpr* mice, such as FB (18) and FD (19), exhibited significantly decreased glomerular C3 deposition levels, maintained serum C3 levels, and improved glomerular pathological score. Furthermore, Sekine et al. reported the benefit of the selective inhibition of the AP for renal disease in lupus-prone MRL/*lpr* and NZM2410 mice by therapeutic administration of a targeted and selective inhibitor of the AP CR2-fH, compared to CR2-Crry, which inhibits all complement pathways (20, 21).

In contrast to the extensive evidence on the CP and AP, little is known in regard to the involvement of the LP in lupus. Previous human genetic studies in lupus have shown that there are five polymorphic sites in MBL, a recognition molecule for the LP, and are associated with serum levels of MBL and the development of lupus (22–24). Villareal et al. reported that Spanish patients carrying a genetic variant in codon 54 of MBL, one of the previously reported five variants, had a high risk of developing lupus (25). Furthermore, Seelen et al. (26) supported the data described above by investigating the LP activation and several serum auto-Ab levels in lupus patients with all five MBL variants. They indicated that patients carrying MBL variant alleles exhibited impaired serum activation levels of the LP, increased serum anti-cardiolipin and anti-C1q antibodies, and similar levels of serum MBL when compared to patients without those variant alleles. They concluded that the mutant MBL may have low-binding activity to apoptotic cells, and result in defective clearance of auto-Ags, suggesting a protective role for MBL or the LP in auto-Ab production in lupus. Recently, Sato et al. reported that lupus patients with MBL/L-ficolin and properdin deposition in their glomeruli had significantly higher urinary protein excretion levels compared to patients without glomerular deposition, suggesting pathogenic and exacerbating roles for the LP and AP in the development of lupus glomerulonephritis (27). Collectively, whether the role of the LP in lupus pathogenesis is protective or exacerbating remains unclear. Also, direct evidence on the role of the LP in SLE has yet to be demonstrated. In this context, an association between the LP and SLE that involves complement activation is assumed and should be elucidated.

Since total blockade of all complement pathways is apparently not appropriate as a therapeutic strategy for SLE, an individual role for the three different complement pathways should be clarified. In the present study, we generated lupus-prone MRL/*lpr* mice that were genetically deficient for MASP-1/3 by using a backcrossing strategy and confirmed that their sera lacked both LP and AP complement activity. We then analyzed their lupus-like disease serologically and renal pathologically.

## Materials and Methods

### Mice

MRL/MpJ-*Fas^lpr^*/J (MRL/*lpr*; stock no. 000485) was purchased from The Jackson Laboratory. *Masp1/3^−/−^* C57BL/6 mice (4) were backcrossed for seven generations with MRL/*lpr* mice to generate an eighth backcross generation of *Masp1/3^+/−^* MRL/*lpr* mice. For efficient and precise backcrossing, each generation of backcrossed offspring was checked for 12 microsatellite markers corresponding to disease-susceptibility regions (*D4Mit12, D4Mit17, D5Mit13, D5Mit24, D5Mit145, D7Mit39, D7Mit57, D7Mit211, D10Mit11, D10Mit20, D17Mit16*, and *TNF*) (28), and the *Fas* genotype by PCR with genomic DNA was used as a template. The eighth generation was bred to yield *Masp1/3^+/+^, Masp1/3^+/−^*, and *Masp1/3^−/−^* MRL/*lpr* mice. All animal experiments that included housing, breeding, and using the mice were reviewed and approved by the Animal Experiments Committee of Fukushima Medical University (approval no. 26001 and 28012), and were performed in accordance with the guidelines for the care and use of laboratory animals established by the Committee.

### Assays for C4 Deposition Onto Mannan-Coated Microtiter Plates

The LP activity in sera was determined by C4 deposition assay onto a mannan-coated microplate according to a method by Takahashi et al. (4). A Nunc 96-well optical bottom plate was coated with 100 µL of 10 µg/mL mannan (Sigma-Aldrich) diluted in 50 mM Na-carbonate/bicarbonate buffer (pH 9.5) by overnight incubation at 4°C. The wells were blocked with 1.0% BSA in Tris-buffered saline (TBS) containing 0.1% Tween-20 and 5 mM CaCl_2_ (TBST/Ca) for 1 h at room temperature (RT). After washing three times with TBST/Ca, serially diluted serum samples in TBS/Ca buffer were added to each well and incubated for 1 h at RT. After washing three times with TBST/Ca, diluted purified human C4 (5 µg/mL in TBS/Ca) was added to each well, and incubated for 30 min at 4°C. After washing three times with TBST/Ca, horseradish peroxidase (HRP)-conjugated anti-human C4 Ab (MP Biomedicals) was added to each well. After incubation for 1 h at RT, a TMB Microwell Peroxidase Substrate 2-Component System (Kirkegaard & Perry Laboratories) was added to each well and incubated for 10 min at RT in the dark for color development. An equal volume of 1 M phosphoric acid was added to the substrate solution to stop the color development of TMB, and then the absorbance at 450 nm was measured by a spectrophotometer DTX880 (Beckman Coulter). The LP activity in the TBS/Ca buffer was also measured as a blank experiment, and the activity in sera was expressed as the difference between A_450_ in the serum samples and that in the blank experiment.

### Assays for C3 Deposition Onto Zymosan-Coated Microtiter Plates

The AP activity in sera was determined by zymosan assay according to a method by Takahashi et al. (8). A Nunc 96-well optical bottom plate (Nunc) was coated with 100 µL of 20 µg/mL zymosan (Sigma-Aldrich) suspended in 50 mM Na-carbonate/bicarbonate buffer (pH 9.5) by overnight incubation at 4°C. The wells were blocked with 1.0% BSA in phosphate-buffered saline containing 0.1% Tween-20 (PBST) for 1 h at RT. After washing three times with PBST, serially diluted serum samples in BBS buffer (0.2 M boric acid, 0.14 M NaCl, pH 8.0) supplemented with Mg^2+^-EGTA were added to each well and incubated for 1 h at RT. The wells were then washed with PBST three times, and HRP-conjugated anti-mouse C3 polyclonal antibodies was added to each well and incubated for 1 h at RT, followed by the color development of TMB. The AP activity in BBS buffer was also measured as a blank experiment, and the activity in sera was expressed as the difference between A_450_ in the serum samples and that in the blank experiment.

### Immunoprecipitation and Western Blotting for FD

Mouse sera appropriately diluted in TBS were incubated for 1 h at 4°C with 2.5 µg of affinity-purified rabbit anti-FD IgG and 10 µL of protein A-agarose (GE Healthcare). The beads were washed four times with TBS, and then denatured at 100°C for 5 min in 40 µL of TBS containing 0.1% SDS and 50 mM β-mercaptoethanol. Denatured samples were mixed with 2 µL of 10% Triton X-100 and 0.2 µL of *N*-glycosidase F (Merck Millipore) and incubated for 2 h at 37°C. After centrifugation, supernatants were separated by SDS-PAGE and analyzed by western blotting. FD and pro-FD were detected with peroxidase-conjugated affinity-purified rabbit anti-FD raised in rabbit (8). Images were visualized by chemiluminescence with an ECL Prime Western Blotting Detection Reagent (GE Healthcare) according to the manufacturer’s instruction.

### Determination of Serum Anti-Double-Stranded DNA IgG Levels

As previously described by Gilkeson et al. (29), Nunc 96-well optical bottom plate was coated with 100 µL of 1 µg/mL S1 nuclease-digested calf thymus DNA diluted in 1 × SSC (0.3 M sodium citrate, 0.03 M NaCl, pH 7.0) by overnight incubation at 37°C. Wells were blocked with 1% BSA in PBST (BSA-PBST) for 1 h at RT. After washing three times with PBST, 1/100-diluted serum samples were added to each well, and incubated for 1 h at RT. After washing three times with PBST, bound anti-dsDNA IgG was detected with HRP-conjugated goat anti-mouse IgG (γ-chain specific; Sigma) followed by the color development of TMB. Serum anti-dsDNA IgG levels were expressed as the difference between A_450_ in the serum samples and that in the blank experiment.

### Determination of Serum Circulating IC Levels

Serum circulating IC levels were determined by ELISA according to a method by Stanilova and Slalov (30) with some modifications. A Nunc 96-well optical bottom plate was coated with 100 µL of 1 µg/mL goat anti-mouse C3 Ab (MP biomedicals) diluted in 50 mM Na-carbonate/bicarbonate buffer (pH 9.5) by overnight incubation at 4°C. The wells were blocked with 1% BSA in 50 mM Tris–HCl (pH 7.2) supplemented with 0.05% Tween-20 (Tris–HCl/tw) for 20 min at RT. After washing with Tris–HCl/tw, serially diluted serum samples were added to the wells, and incubated for 1 h at RT. After washing three times with Tris–HCl/tw, bound circulating IgG-IC was detected with HRP-conjugated goat anti-mouse IgG Ab (γ-chain specific; Sigma) followed by the color development of TMB. Serum circulating IgG-IC levels were expressed as the difference between A_450_ in the serum samples and that in the blank experiment.

### Determination of Serum C3 Levels

A Nunc 96-well optical bottom plate was coated with 100 µL of 1 µg/mL goat anti-mouse C3 polyclonal Ab (MP biomedicals) diluted in 50 mM Na-carbonate/bicarbonate buffer (pH 9.5). The wells were blocked with BSA-PBST for 20 min at RT. After washing with PBST, serially diluted serum samples were added to the wells and incubated for 1 h at RT. After washing three times with PBST, bound C3 was detected with HRP-conjugated anti-mouse C3 polyclonal Ab (MP biomedicals) followed by the color development of TMB. The mouse complement C3 calibrator (Kamiya biomedical company) was used to determine the serum C3 concentration.

### Evaluation of Albuminuria

Urine samples were collected every 2 weeks beginning at 12 weeks of age by placing the mice in metabolic cages for 24 h. Urine collection was performed with ampicillin, chloramphenicol, and gentamycin in the bottom of a collection tube to prevent bacterial growth. A Nunc 96-well optical bottom plate was coated with 100 µL of 2.5 µg/mL rabbit anti-mouse albumin polyclonal antibody (MP biomedicals) in 50 mM Na-carbonate/bicarbonate buffer (pH 9.5). Wells were blocked with BSA-PBST for 1 h at RT. After washing with PBST, serially diluted urine samples in BSA-PBST were added to the wells, and incubated for 1 h at RT. After washing three times with PBST, bound urinary albumin was detected with HRP-conjugated anti-mouse albumin polyclonal antibody in BSA-PBST followed by the color development of TMB. Mouse albumin was used as a standard. Urinary albumin excretion was expressed as milligrams of albumin per mouse per day.

### Assessment of Renal Pathology

*Masp1/3^+/+^* and *Masp1/3^−/−^* MRL/*lpr* mice were sacrificed at 24 weeks of age for pathological evaluation. At the time of sacrifice, kidneys were recovered and divided into two sections. One section was placed in buffered formalin for subsequent embedding in paraffin, sliced, and stained with H&E. The H&E-stained slides were assessed *via* light microscopy for glomerular (glomerular inflammation, proliferation, thickness basement membrane, epithelial reactivity, crescent formation, and necrosis) and interstitial pathologies. Pathological scores from 0 to 4+ (0, none; 1+, mild; 2+, moderate; 3+, moderate to severe; 4+, severe) were assigned for each of these features and then added together to yield a final score, and an overall glomerular score was derived.

### Immunofluorescence Staining

The other kidney section from *Masp1/3*^+/+^ and *Masp1/3^−/−^* MRL/*lpr* mice was embedded in an OCT compound and frozen in liquid nitrogen for cryosections. The frozen sections were cut into 5-µm slices, fixed with acetone, and stained with FITC-conjugated antibodies: goat anti-mouse IgG, C1q, MBL-A, MBL-C, Ficolin-A, Ficolin-B, and C3 diluted 1:100. The severity of glomerulonephritis and IC deposition was determined in a blind manner. Scores ranged from 0 to 4+, where 0 corresponded to a non-autoimmune healthy mouse and 4+ to the maximal alteration observed in the study.

### Determination of Serum Urea Nitrogen (UN) Levels

Serum UN was determined using a UN Colorimetric Detection Kit (Arbor Assays) according to the manufacturer’s instructions. Serum from BALB/c mice was subjected to the assay as an indicator for a normal level of serum UN.

### Statistical Analysis

Statistical analysis was performed using a GraphPad Prism 6 software for Mac OS X (GraphPad Software, San Diego, CA, USA). Single groups were compared using an unpaired two-tailed *t* test.

## Results

### Ability of LP and AP Activation in Sera From MRL/*lpr* Mice

**Figure 1:**
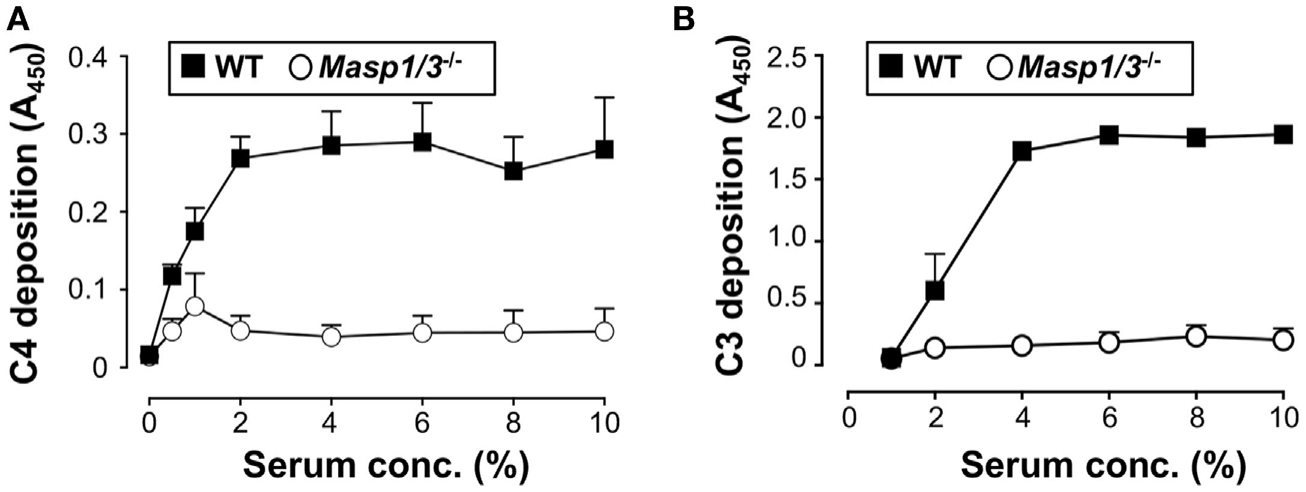
Inability of the lectin pathway **(A)** and alternative pathway **(B)** activation in sera from 10- to 14-week-old *Masp1/3^−/−^* MRL/*lpr* mice. Values are means ± SD (*n* = 3).

Our group had previously demonstrated that mice deficient for MASP-1/3 had little-to-no activation of both the LP and AP with an inactive form of FD in their sera at the C57BL/6 background (8). In the current study, we first tested if lupus-prone MRL/*lpr* mice also had altered activation of the LP and AP in MASP-1/3-deficient serum. To assess the activity of the LP and AP in MRL/*lpr* mice, we performed the C4 deposition assay with mannan-coated plates and C3 deposition assay with zymosan-coated plates. As expected, sera from *Masp1/3^+/+^* wild-type MRL/*lpr* mice had C4 deposition activity on the mannan-coated plates and C3 deposition activity on the zymosan-coated plates in a dose-dependent manner (Figure 1). In contrast, both C4 and C3 deposition from the sera of *Masp1/3^−/−^* MRL/*lpr* mice were significantly lower than those in the wild-type MRL/*lpr* mice. These results indicate that MASP-1 and/or MASP-3 is involved in the activation of the LP and AP in MRL/*lpr* mice as in C57BL/6 mice.

**Figure 2:**
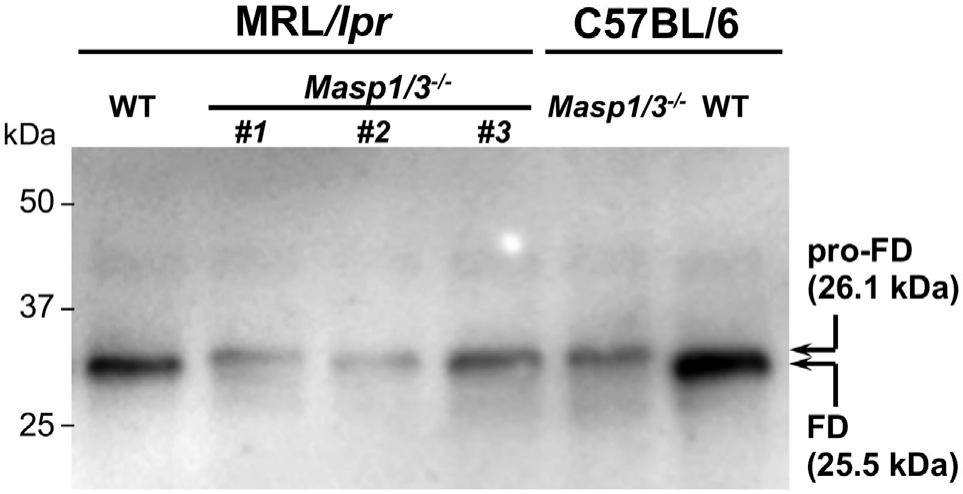
Immunoblotting for factor D (FD) in sera from 10- to 14-week-old MRL/*lpr* and C57BL/6 mice. Serum FD were purified by immunoprecipitation with a rabbit anti-mouse FD antibody, deglycosylated with *N*-glycosylase F, separated by SDS-PAGE, and blotted onto a PVDF membrane. Purified pro-FD (26.1 kDa) and FD (25.5 kDa) were detected with a horseradish peroxidase-conjugated rabbit anti-mouse FD antibody. Consistent with our previous report on C57BL/6 mice (8), serum FD in *Masp1/3^−/−^* MRL/*lpr* mice was in an inactive form (pro-FD) when tested for serum samples from three independent *Masp1/3^−/−^* MRL/*lpr* mice (#1–3). In contrast, serum FD in WT MRL/*lpr* mice was in an active form as well as in WT C57BL/6 mice.

It has been shown that circulating FD in *Masp1/3^−/−^* C57BL/6 mice is a zymogen (inactive form of FD or pro-FD) that can be distinguished from the active form of FD by the difference in molecular weight, as activated FD lacks N-terminal peptide QPRGR, which pro-FD possesses (8). As shown in Figure 2, the deglycosylated FD in the *Masp1/3^−/−^* mice both at the MRL/*lpr* and C57BL/6 background was slightly larger than that in the wild-type mice. These results demonstrate that lupus-prone MRL/*lpr* mice deficient for MASP-1/3 had little-to-no activation of both the LP and AP with an inactive form of FD in sera.

### Serum Levels of IgG Anti-dsDNA Auto-Abs, Circulating ICs and C3 in MRL/*lpr* Mice

**Figure 3:**
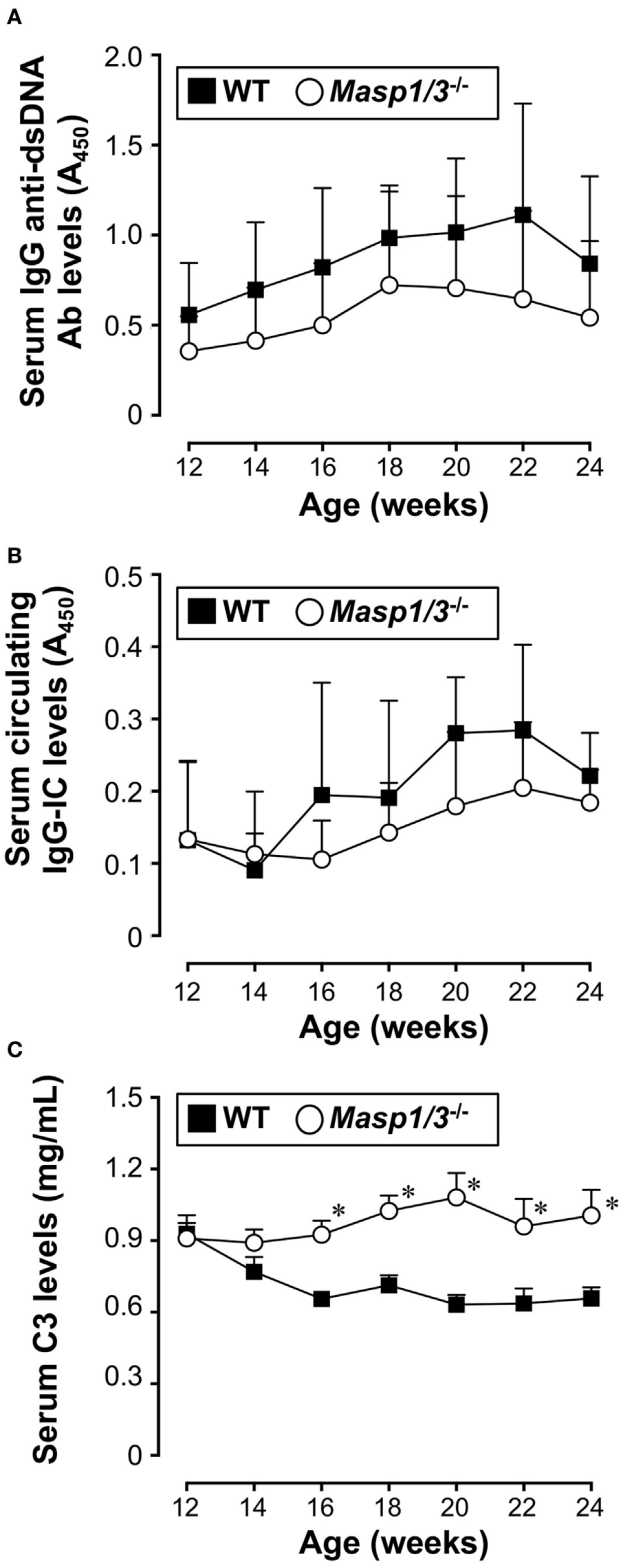
Serum levels of IgG anti-dsDNA Ab **(A)**, circulating IgG-immune complex (IC) **(B)**, and C3 **(C)** in 12- to 24-week-old MRL/*lpr* mice. Values are means ± SD (*n* = 6–8). Values with asterisks are significantly different at *p < 0.05 against values from age-matched WT MRL/*lpr* mice.

Production of IgG anti-dsDNA auto-Ab is strongly associated with lupus-like renal disease in MRL/*lpr* mice. To assess the effect of MASP-1/3 deficiency in anti-dsDNA Ab production, we measured the serum levels of IgG anti-dsDNA Ab in MRL/*lpr* mice by ELISA starting from 12 to 24 weeks of age. As shown in Figure 3A, there was a progressive rise in serum anti-dsDNA Ab levels both in the wild-type and *Masp1/3^−/−^* MRL/*lpr* mice after 12 weeks of age. There was a trend toward lower serum anti-dsDNA Ab levels in the *Masp1/3^−/−^* MRL/*lpr* mice compared to the wild-type MRL/*lpr* mice; however, the difference did not reach statistical significance at any point in time until 24 weeks of age. These results indicate that there was minimal or no impact of MASP-1/3 deficiency on serum auto-Ab levels in MRL/*lpr* mice.

We next assessed serum levels of circulating IgG-ICs in MRL/*lpr* mice by anti-C3 anti-mouse IgG sandwich ELISA starting from 12 weeks of age. As shown in Figure 3B, there was no statistically significant difference between the groups at any point in time until 24 weeks of age.

Serum C3 levels in patients with SLE is known to show an inverse correlation with disease activity due to its consumption following activation of the complement cascade. We measured serum C3 levels in the MRL/*lpr* mice starting from 12 weeks of age. Those levels in the wild-type MRL/*lpr* mice decreased as the mice aged and showed an inverse correlation with serum IgG anti-dsDNA Ab levels (Figure 3C). In contrast, the serum C3 levels in the *Masp1/3^−/−^* MRL/*lpr* mice were maintained at constant levels, and were significantly higher than those in the wild-type MRL/*lpr* mice at 16 weeks of age, and continued to be higher until the time of sacrifice (Week 24). These results show that there was a significant effect of MASP-1/3 deficiency on the reduction of serum C3 consumption in MRL/*lpr* mice.

### Albuminuria in MRL/*lpr* Mice

**Figure 4:**
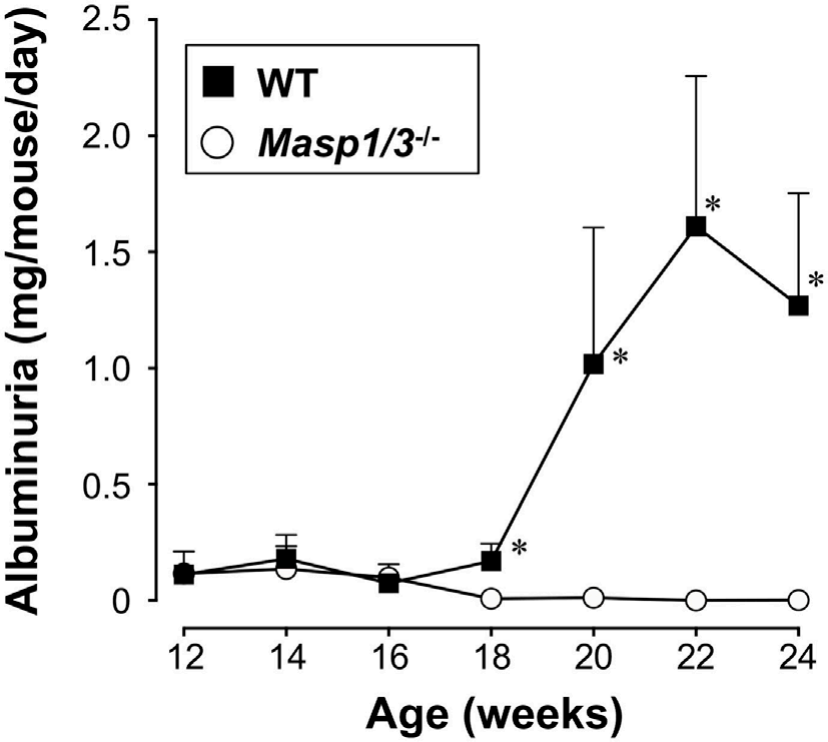
Urinary albumin excretion levels in 12- to 24-week-old MRL*/lpr* mice. Values are means ± SD (*n* = 6–8). Values with asterisks are significantly different at *p < 0.05 against values from age-matched wild-type MRL/*lpr* mice.

Urinary albumin or protein excretion in SLE reflects glomerular damage to the charge/size barrier between the capillary lumen and urinary space, and, therefore, through the glomerular capillary endothelial cells, glomerular basement membrane, and podocytes in glomeruli. To evaluate the effect of MASP-1/3 deficiency on renal function, we measured 24 h of urinary albumin excretion levels of MRL/*lpr* mice, starting at 12 weeks of age. As shown in Figure 4, the wild-type MRL/*lpr* mice developed a high level of albuminuria after 18 weeks of age. In contrast, the MRL/*lpr* mice deficient for MASP-1/3 had significantly less albuminuria remaining at less than 0.1 mg/mouse/day during the tested period compared to wild-type littermates. These results suggest a significant role for MASP-1/3 in the development of glomerular disease in MRL/*lpr* mice.

### Glomerular Deposition of ICs and Complement in MRL/*lpr* Mice

**Figure 5:**
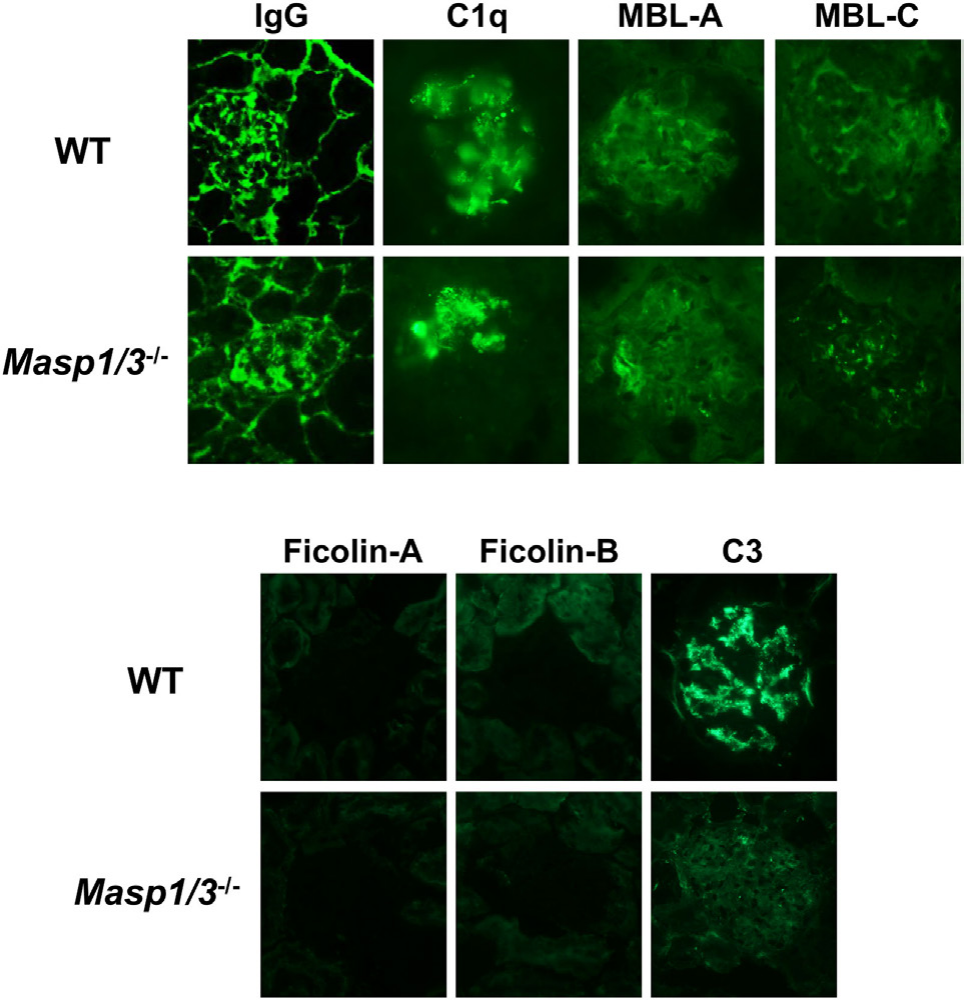
Assessment of glomerular depositions of IgG and complement components. Glomerular depositions of IgG, C1q, MBL-A, MBL-C, Ficolin-A, Ficolin-B and C3 were evaluated using kidney sections from 24-week-old WT and *Masp1/3^−^*^/^*^−^* MRL/*lpr* mice. Similar results were obtained from five independent mice in each group, and representative images were shown here. Original magnification 400×.

To determine the mechanistic effect of the absence of MASP-1/3 on the reduction of albuminuria observed in MRL/*lpr* mice, mice were sacrificed at 24 weeks of age, and their kidneys were recovered for pathological analysis. To assess glomerular ICs (IgG), C1q, MBL-A, MBL-C, Ficolin-A, Ficolin-B, and C3 deposition, frozen kidney sections were stained with fluorescein-conjugated Abs against mouse IgG, C1q, MBL-A, MBL-C, Ficolin-A, Ficolin-B, or C3. There was no significant difference in glomerular IgG, C1q, MBL-A, or MBL-C deposition levels between wild-type and *Masp1/3^−/−^* MRL/*lpr* mice, while glomerular deposition levels of Ficolin-A and Ficolin-B were low or undetectable in these mice (Figure 5; Table 1). It was indicated that wild-type and *Masp1/3^−/−^* MRL/*lpr* mice had similar deposition levels of the CP and LP recognition molecules in their glomeruli. Importantly, glomerular C3 deposition was readily evident in wild-type MRL/*lpr* mice, while *Masp1/3^−/−^* MRL/*lpr* mice had significantly reduced levels of glomerular C3 deposition compared to their wild-type littermates. These results suggest that acceleration of the LP and/or AP activation, which is mediated by MASP-1/3, rather than CP activation, plays an important role in C3 consumption in murine lupus.

**Table 1:** Scores for glomerular deposition of IgG and complement in 24-week-old MRL/*lpr* mice.

Genotype	IgG	C1q	Mannose-binding lectin (MBL)-A	MBL-C	Ficolin-A	Ficolin-B	C3
WT	1.6 ± 1.0	1.4 ± 1.2	2.2 ± 1.2	2.4 ± 1.1	n.d.	n.d.	2.5 ± 0.6
Masp1/3^−/−^	1.7 ± 1.0	0.9 ± 0.6	2.2 ± 0.7	2.7 ± 0.7	n.d.	n.d.	1.3 ± 0.7*

All glomerular immunofluorescence images were interpreted by three independent persons in a blinded manner, and graded from 0 to 4+ (0, none; 1+, mild staining; 2+, moderate staining; 3+, moderate-high staining; 4+, high staining) for fluorescence intensity. All values are means ± SD (*n* = 5). Values with asterisks were significantly different at *p < 0.05 against values from age-matched WT mice. n.d., not detected.

### Renal Pathology in MRL/*lpr* Mice

**Figure 6:**
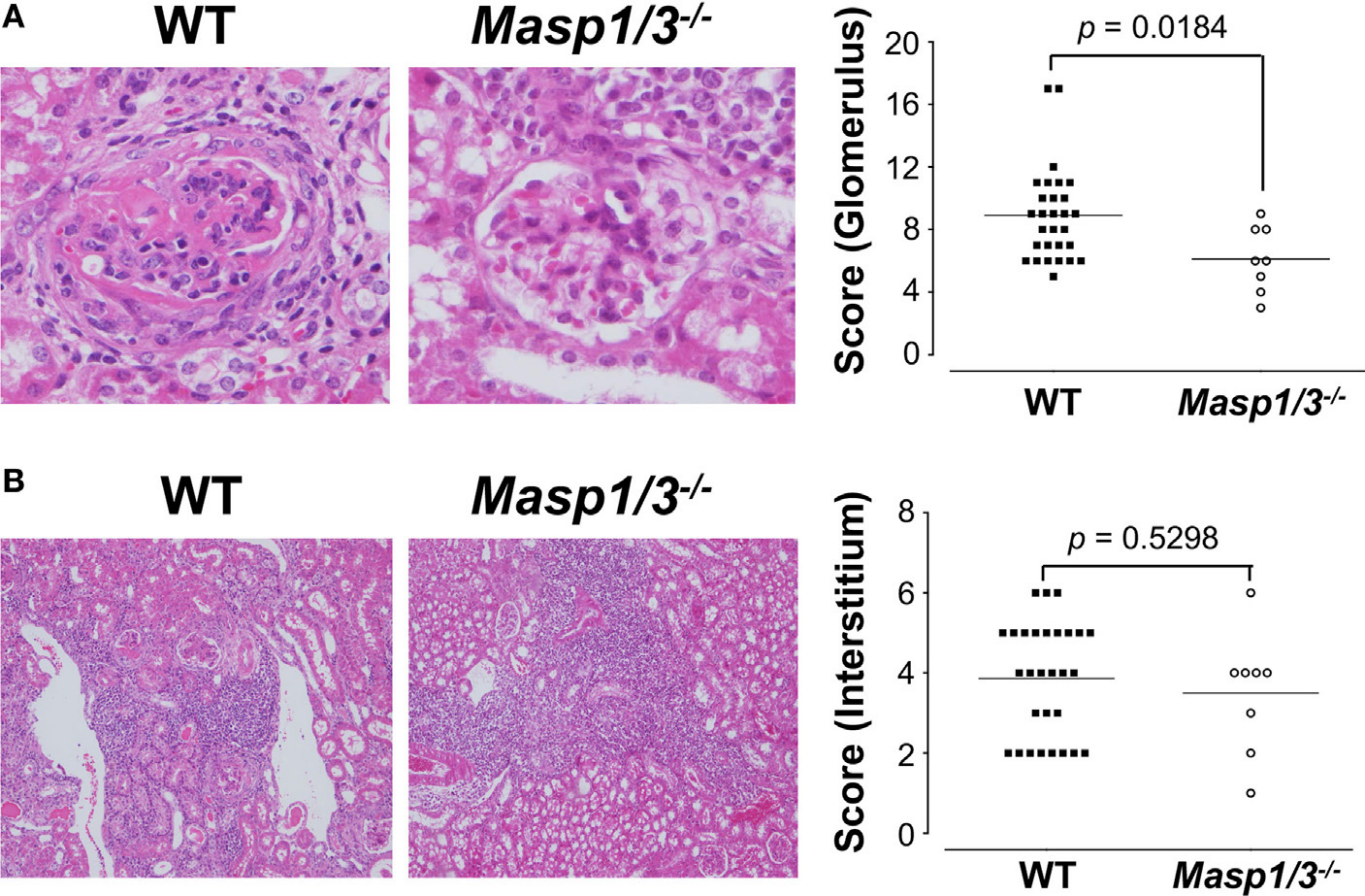
Assessment of glomerular **(A)** and renal interstitial **(B)** pathologies stained with hematoxylin and eosin. Glomerular pathology was graded from the sum of scores for glomerular inflammation, thickness of basement membrane, epithelial cell reactivity, crescent formation, and necrosis. Interstitial pathology was graded using the sum of scores for perivascular inflammation and inflammatory cell infiltration. Scores were graded as 0 to 4+ (0, none; 1+, mild; 2+, moderate; 3+, moderate-high; 4+, high). Original magnification 200× for glomerular pathology, and 40× for interstitial pathology.

Kidney sections were stained with H&E and assessed by histological scoring for overall glomerular proliferative changes, crescent formation and necrosis, and interstitial inflammation. As expected, the wild-type MRL/*lpr* mice exhibited diffuse glomerulonephritis, including cellular proliferation, inflammation, glomerular expansion, fibrocellular crescents, and interstitial inflammation (Figure 6A). The *Masp1/3^−/−^* MRL/*lpr* mice, however, had significantly less pathological features of glomerular disease compared to their wild-type littermates, with a reduction in mesangial expansion, glomerular inflammation, focal hypercellularity, and crescent formation (reflected in the renal score, Figure 6A, *p* < 0.05). On the other hand, both strains exhibited renal interstitial inflammation with no significant difference in disease score between the two groups (Figure 6B). These results indicate that MASP-1/3 is intimately associated with progression in glomerulonephritis, but not with renal interstitial inflammation in MRL/*lpr* mice.

### Serum UN Levels in *Masp1/3^−/−^* MRL/*lpr* Mice

**Figure 7:**
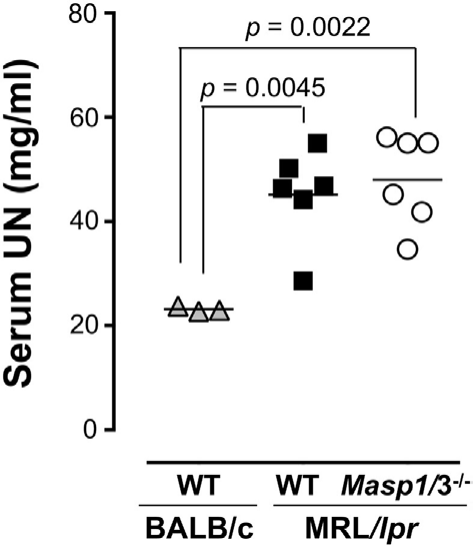
Serum urea nitrogen (UN) levels in MRL/*lpr* mice. UN levels were measured using sera from WT and *Masp1/3^−^*^/^*^−^* MRL/*lpr* mice at the age of 24 weeks (*n* = 6 in each group). Sera from 25-week-old WT BALB/c mice (*n* = 3) were used as a healthy control.

To evaluate renal function, serum UN levels of *Masp1/3^+/+^* and *Masp1/3^−/−^* MRL/*lpr* mice were measured using serum obtained at 24 weeks of age. As shown in Figure 7, serum UN levels of *Masp1/3^+/+^* MRL/*lpr* and *Masp1/3^−/−^* mice were significantly higher than those of non-autoimmune BALB/c mice, while there was no statistically significant difference of the serum UN levels between *Masp1/3^+/+^* and *Masp1/3^−/−^* MRL/*lpr* mice.

### Mortality of MRL/*lpr* Mice

**Figure 8:**
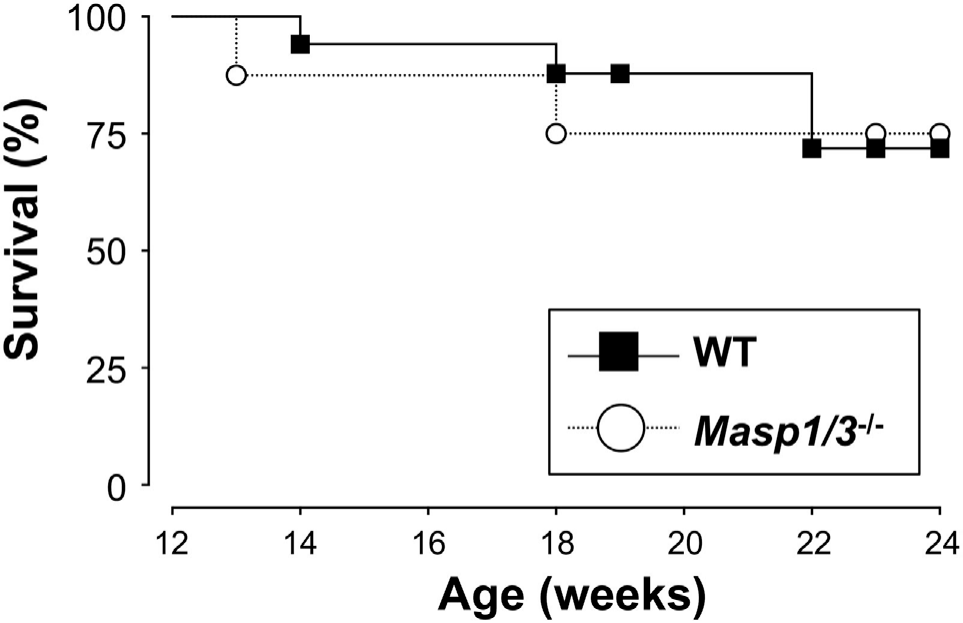
Survival curves for WT (*n* = 17) and *Masp1/3^−/−^* (*n* = 8) MRL/*lpr* mice. Mortality was recorded from 12 weeks old to the time at which the mice were sacrificed (24 weeks).

Despite having significantly less albuminuria and pathological features of glomerular disease in the *Masp1/3^−/−^* MRL/*lpr* mice, the absence of MASP-1/3 had no significant beneficial effect on survival in the MRL/*lpr* mice until the time of sacrifice (week 24) (Figure 8).

## Discussion

To determine the role of MASP-1/3 in lupus nephritis, we backcrossed MASP-1/3-deficient C57BL/6 mice into lupus-prone MRL/*lpr* mice for eight generations, and then intercrossed the *Masp1/3^+/−^* mice. The results presented in this report indicate that the absence of MASP-1/3 in MRL/*lpr* mice has a significant effect on the activation of the complement and development of lupus-like glomerulonephritis. Consistent with previous results in *Masp1/3^−/−^* C57BL/6 mice (4, 8), the MASP-1/3-deficient MRL/*lpr* mice of the current study had little-to-no activation of the LP and AP, and significantly reduced glomerular C3 deposition, albuminuria and pathologic renal scores compared with their wild-type MASP-1/3-producing littermates.

To date, mice deficient for different complement components have been generated, and the roles for each complement component in the development of lupus or lupus-like disease have been analyzed. In lupus nephritis, activation of the complement system is thought to be triggered via the CP. This is because the presence of autoantibodies is a requirement for the development of lupus nephritis (31), and deposition of complement proteins including the CP components C1q, C4, and C3 in the glomeruli are key features of lupus nephritis. However, as summarized in Table 2, mice deficient for C1q or C4 showed high antinuclear antibody titers, anti-DNA autoantibody levels, and glomerulonephritis with impairment in the clearance of apoptotic cells (15–17, 32). Although the severity of serum autoantibody levels or glomerular disease is genetic background-dependent, C1q and C4, the complement components of the CP, provide an important protective role against the development of lupus or lupus-like glomerulonephritis in mice. This observation is consistent with patients with homozygous genetic deficiencies of an early component of the CP (i.e., C1q, C1r, C1s, C4A/C4B, and C2), which are strongly associated with the risk of developing SLE or a lupus-like disease (33). As for C3, the converging point for activation of all three complement pathways, *C3^−/−^* mice in (129 × C57BL/6)*lpr* background exhibited no difference in serum autoantibody levels, glomerular IgG deposition levels or glomerular pathological scores compared to *C3^+/+^* (129 × C57BL/6)*lpr* mice (16). Similarly, C3-deficient lupus-prone MRL/*lpr* mice exhibited no difference in serum autoantibody levels, glomerular pathological scores, or survival, but had significantly increased levels of glomerular IgG deposition and albuminuria (34). These results suggest that C3 plays a beneficial role in lupus-like glomerular disease *via* clearance of ICs. Indeed, there is an association of inherited human C3 deficiency with IC-related disorders, including membranoproliferative glomerulonephritis, SLE, and vasculitis (35).

**Table 2:** Effects of genetic deficiency for complement components on lupus-like disease in murine models of lupus.

Knockout genes	Classical pathway components						Alternative pathway (AP) components		LP and AP components
		
	C1q		C4		C3		Factor B	Factor D	Mannose-binding lectin-associated serine protease-1/3
Strain	129/Ora × C57BL/6	MRL^**+**/**+**^	B6, 129 × C57BL/6, BALB/c × 129 × C57BL/6	(129/Sv × C57BL/6)*lpr*	(129/Sv × C57BL/6)*lpr*	MRL/*lpr*	MRL/*lpr*	MRL/*lpr*	MRL/*lpr*

H2 haplotype	H2^b/b^	H2^k/k^	H2^b/b^	H2^b/b^	H2^b/b^	H2^k/k^	H2^b/b^	H2^k/k^	H2^k/k^

Antinuclear antibody titer	Increased (55%)	Increased		Increased	No difference				
Serum anti-DNA levels		Increased	Increased	Increased	No difference	No difference	Decreased	No difference	No difference
Serum C3 levels							Increased	Increased	Increased
Glomerular IgG deposits			Increased	Increased	No difference	Increased	Decreased	No difference	No difference
Glomerular C3 deposits							Decreased	Decreased	Decreased
Proteinuria			No difference			Increased	Decreased	No difference	Significantly decreased
Glomerulo-nephritis (renal score)	Developed (25%)	Developed	No difference	Developed	No difference	No difference	Improved	Improved	Improved
	Multiple apoptotic cell bodies and immune deposits			Hypercellularity, glomerular enlargement, mesangial thickening					
Survival	Decreased	Decreased				No change	Improved	No change	No change

Reference	(15)	(17)	(32)	(16)	(16)	(34)	(18)	(19)	This study

*The blue, green, yellow, or orange shades represent phenotypes of mice lacking complement component of the classical pathway, C3, the alternative pathway, or the alternative pathway and lectin pathway, respectively. Red or blue letters indicate that the corresponding complement component defects are pathogenic or protective against the development of murine lupus-like disease, respectively*.

*CP, classical pathway; AP, alternative pathway; LP, lectin pathway; MASP-1/3, mannose-binding lectin-associated serine protease-1/3; ANA, anti-nuclear antibody*.

In contrast to the complement component of the CP and C3, no association of the deficiencies of the complement factors of the AP with the risk of developing lupus in humans has been reported, suggesting at least that there are no protective roles for the AP complement factors against the development of lupus. To address the question whether the AP complement factors play protective or exacerbating roles in the development of lupus, MRL/*lpr* mice deficient for the AP complement factors, FD or FB, were generated, and their lupus-like disease was analyzed. As summarized in Table 2, both the FB- and FD-deficient MRL/*lpr* mice exhibited reduced glomerular C3 deposition levels, maintained serum C3 levels, and improved glomerular pathological scores compared to the wild-type MRL/*lpr* mice (18, 19). Interestingly, the MRL/*lpr* mice exhibited somewhat different disease phenotypes between *FB^−/−^* and *FD^−/−^* genetic backgrounds. Similar to the *FB^−/−^* MRL/*lpr* mice, the *FD^−/−^* MRL/*lpr* mice exhibited maintained serum C3 levels, significantly reduced glomerular C3 deposition levels and improved pathological renal scores. However, the *FB^−/−^* MRL/*lpr* mice exhibited some additional beneficial effects on reduced levels of serum anti-DNA antibody and glomerular IgG deposition, proteinuria, as well as reduced IgG3 cryoglobulin production and renal vasculitis (18). We hypothesize that these additional improvements observed in the *FB^−/−^* MRL/*lpr* mice are unlikely due to the absence of FB itself but likely due to MHC-linked effects. MRL/*lpr* mice deficient for the *FB* gene, which was located on the MHC class III region, were carrying the H2^b/b^ MHC haplotype, whereas wild-type or *FB^+/+^* MRL/*lpr* mice were carrying the H2^k/k^ MHC haplotype (18). These differences perhaps affect Ag presentation leading to differences in pathogenic autoantibody production or immunoglobulin isotype switching. In addition, the MHC-linked effects on MRL/*lpr* mice were tested by generating H2^b/b^ *FB^+/+^* congenic MRL/*lpr* mice (36, 37). Those studies showed that, similarly to H2^b/b^ *FB^−/−^* MRL/*lpr* mice, 70% of H2^b/b^ *FB^+/+^* MRL/*lpr* mice spontaneously developed serum IgG3 deficiency with reduced anti-dsDNA autoantibody levels and exhibited significantly reduced albuminuria compared to wild-type (H2^k/k^ *FB^+/+^*) MRL/*lpr* mice. Production of IgG3 in MRL/*lpr* mice is one of the major factors responsible for the development of glomerulonephritis in such mice (38). Therefore, H2^b/b^ haplotype-linked IgG3 deficiency accounts for at least part of the reduced proteinuria observed in H2^b/b^ *FB^−/−^* MRL/*lpr* mice.

In the present study, the *Masp1/3^−/−^* MRL/*lpr* mice, lacking the LP and AP, had significantly reduced glomerular C3 deposition, pathological glomerular disease and albuminuria compared to their wild-type littermates. Similarly, the *FD^−/−^* MRL/*lpr* mice had significantly reduced glomerular C3 deposition and improved renal pathology but had no protective effect on albuminuria. Thus, it is possible that the reduced albuminuria observed in the *Masp1/3^−/−^* MRL/*lpr* mice was in part dependent on the inability of LP activation. Involvement of the LP in the development of glomerulonephritis in human lupus is largely unknown. Previously reported genetic analyses of lupus patients showed that gene polymorphism of MBL, the recognition molecule of the LP, is linked to lupus susceptibility (22–24). A reduced functional activity of the LP, in relation to the expression of MBL variant alleles, is associated with increased levels of serum autoantibodies (26). These observations suggest a beneficial role of MBL in the clearance of apoptotic material that is somewhat similar to the role of the CP recognition molecule C1q. Meanwhile, previously reported pathoclinical analyses of lupus patients demonstrated deposition of the LP recognition molecules in their glomeruli; MBL in 82%, L-Ficolin in 63.6% (39). In that report, patients with glomerular MBL deposition had a higher mean of proteinuria than patients without glomerular MBL deposition, suggesting involvement of the LP in the development of proteinuria. Another study reported that lupus patients with glomerular MBL/L-ficolin and properdin deposition, which is deposits of the LP and AP components, had significantly higher levels of proteinuria than patients without these glomerular depositions (27). Consistent with our results, that report showed the significance of glomerular activation of the LP and AP in lupus nephritis.

In addition to MBL and ficolins, collectin-10 (CL-10 or CL-L1) and collectin-11 (CL-11 or CL-K1) are known to be MASP-1/3-associated collectins (40, 41). Recently, Wu et al. reported a pathogenic role for collectin-11 in the development of tubulointerstitial fibrosis in murine models of renal ischemia-reperfusion injury (42). However, the contribution of collectin-10 and/or collectin-11 to the development of lupus glomerulonephritis is largely unknown. In the present study, we did not evaluate collectin-10 or collectin-11 deposits in glomeruli or tubulointerstitial regions of MRL/*lpr* mice. Further studies are needed to clarify their contribution to the development of renal injury in lupus.

The association of LP serine proteases MASPs (MASP-1, MASP-2, and MASP-3) and MBL-associated proteins MAps (MAp19 and MAp44) with lupus characteristics has also previously been assessed (43). MAp19 and MAp44, the splicing variants of the *Masp2* and *Masp1* genes, respectively, are thought to have a regulatory function in LP activation (44, 45). Interestingly, plasma concentrations in lupus patients were higher than the healthy controls regarding MASP-1, MASP-3, and MAp44, but not MASP-2. These results suggested that the association of MASP-1 and/or MASP-3 with lupus characteristics goes beyond the role of complement activation. Indeed, unlike MASP-2 or MASP-3, MASP-1 has many substrates including non-complement proteins, and the roles of MASP-1 other than in complement activation have been reported. For example, MASP-1 directly digests fibrinogen and factor XIII, playing a role in coagulation (46, 47). Besides the complement and the coagulation system, MASP-1 plays a role in the kinin generation system, where recombinant MASP-1 or natural MBL-MASPs are able to cleave high-molecular-weight kininogen and liberate bradykinin (48). Although the efficiency of MASP-1-mediated cleavage in the kinin generating system is low compared to that of the plasma kallikrein-mediated cleavage, it could be important when the LP activates locally, such as in the glomeruli in lupus patients. Local activation of the LP in glomeruli could lead to local bradykinin production that could contribute to the development of proteinuria. Furthermore, a significant association of MASP-1 in cellular immunity has been reported. MASP-1 can cleave protease activated receptors on the surface of endothelial cells, which results in the pro-inflammatory activation of endothelial cells, followed by local infiltration of neutrophils and endothelial cell damage (49). These tissue damages could also occur on glomerular endothelial cells in lupus patients with glomerular MBL-MASP-1 deposits. Taken together, the absence of MASP-1 in addition to the absence of MASP-3 is highly beneficial to protect lupus patients from the development of glomerulonephritis.

The absence of MASP-1/3 showed significantly reduced glomerulonephritis and albuminuria in the MRL/*lpr* mice of the present study; however, it did not improve their serum BUN levels or survival rate. One possible reason is that, similar to the wild-type MRL/*lpr* mice, the *Masp1/3^−/−^* MRL/*lpr* mice developed tubulointerstitial nephritis. Moreover, elevated serum BUN levels reflect tubulointerstitial damage and progression of renal failure as well as decreased glomerular filtration rates (50, 51). This observation suggests that the mechanism(s) underlying glomerular disease, which is significantly associated with MASP-1/3, are distinct from that of in tubulointerstitial nephritis in this model, and the absence of MASP-1/3 alone may not be enough to improve overall renal function in MRL/*lpr* mice. Another possible reason is that MASP-1/3 plays an important role in ontogenesis. Deficiency of MASP-1/3 or its complex partner CL-K1 leads to the development of 3MC syndrome (52), which is characterized by unusual facial features and problems affecting other tissues and organs of the body. Indeed, the total body weight of the *Masp1/3^−/−^* MRL/*lpr* mice in the current study was significantly lower than that of the age-matched wild-type MRL/*lpr* mice (Figure S1 in Supplementary Material). Therefore, it is possible that *Masp1/3^−/−^* MRL/*lpr* mice, which lack MASP-1/3 congenitally, have lowered resistance against stress-related autoimmune response. The direct effect of MASP-1/3 regarding complement activation on lupus may be elucidated in further studies using anti-MASP-1/3 agents, such as a specific inhibitor and anti-MASP-1/3 Ab, by suppressing MASP-1/3 therapeutically.

In summary, our experiments revealed that MASP-1/3 is required for the development of glomerulonephritis in MRL/*lpr* mice. The major role of MASP-1/3 for glomerular disease in this model is supposed to be in the activation of the AP that is initially activated *via* the CP. In addition, glomerular MBL deposition observed in MRL/*lpr* mice suggests that the activation of the LP, in which MASP-1 plays a significant role, was also involved in their glomerular disease. Moreover, the absence of MASP-1, which also directly plays roles in coagulation, the kinin generation system and endothelial cells, may contribute an additional beneficial effect to protect from glomerular damage. Therefore, the absence of both MASP-1 and MASP-3 is protective against the development of glomerulonephritis in MRL/*lpr* mice. Although further experiments including analysis in different murine lupus strains are required, both proteases are potential therapeutic targets for glomerulonephritis in murine lupus models and possibly in human lupus patients.

## Ethics Statement

All animal experiments that included housing, breeding, and using the mice were reviewed and approved by the Animal Experiments Committee of Fukushima Medical University (approval no. 26001 and 28012), and were performed in accordance with the guidelines for the care and use of laboratory animals established by the Committee.

## Author Contributions

TM and NS are equally credited as first authors in this work: TM, NS, and HS designed the study and wrote the manuscript: TM and NS performed the experiments, analyzed the results, and made figures and tables: YI assisted with the experiments: MT, TF, and HS supervised the study.

## Conflict of Interest Statement

The authors declare that the research was conducted in the absence of any commercial or financial relationships that could be construed as a potential conflict of interest.
