# Supplementary material for: Essential Roles for Mannose-Binding Lectin-Associated Serine Protease-1/3 in the Development of Lupus-Like Glomerulonephritis in MRL/lpr Mice
Source: Front Immunol. 2018 May 28;9:1191. doi: 10.3389/fimmu.2018.01191 (PMC5985374; doi:10.3389/fimmu.2018.01191)
Supplement: Supplementary file 1 [file presentation_1.pptx]

## Slide 1
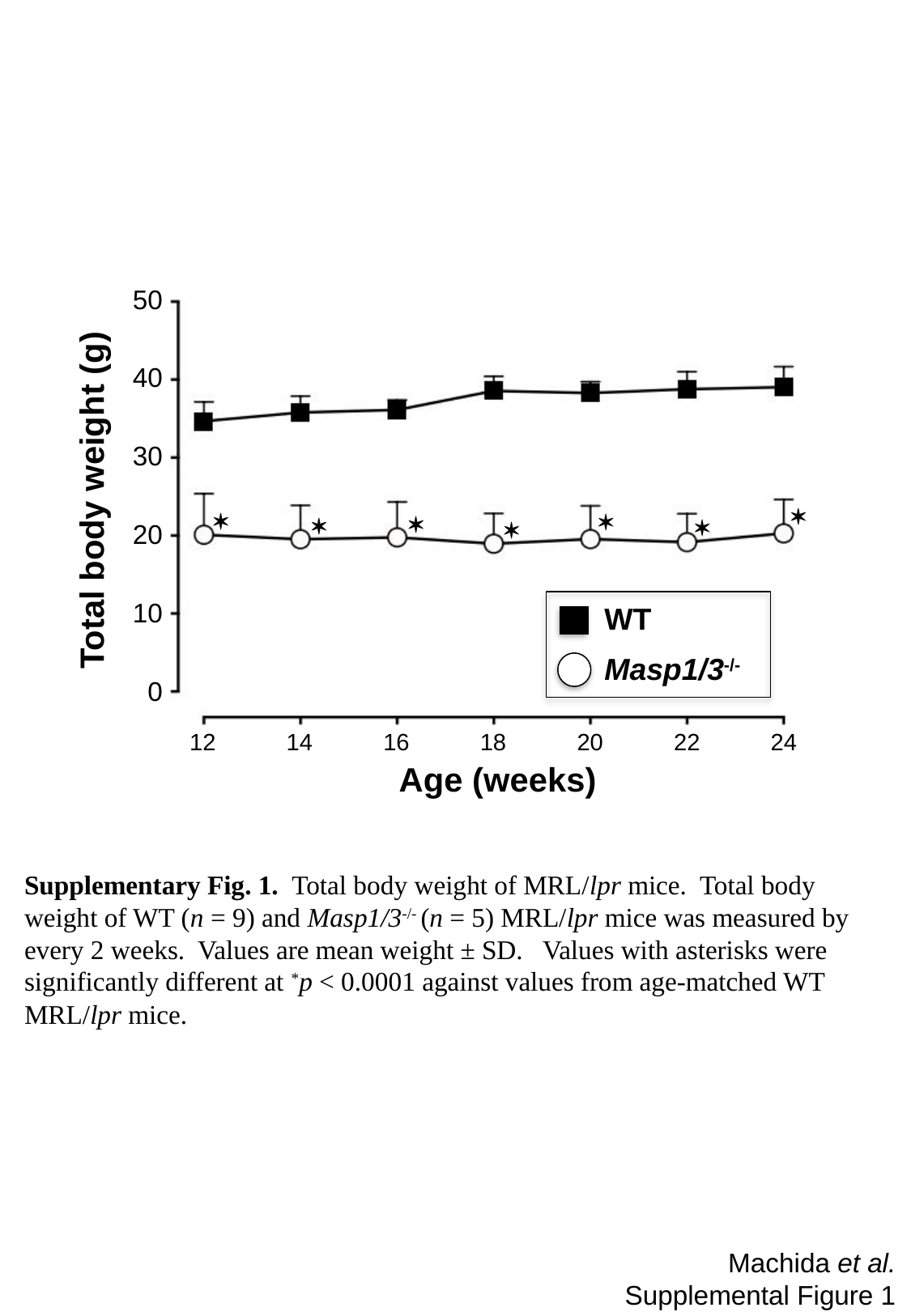

50
40
30
Total body weight (g)
*
*
*
*
*
*
*
20
10
WT
Masp1/3-/-
0
12
14
16
18
20
22
24
Age (weeks)
Supplementary Fig. 1. Total body weight of MRL/lpr mice. Total body weight of WT (n = 9) and Masp1/3-/- (n = 5) MRL/lpr mice was measured by every 2 weeks. Values are mean weight ± SD. Values with asterisks were significantly different at *p < 0.0001 against values from age-matched WT MRL/lpr mice.
Machida et al. Supplemental Figure 1
